# Prevalence of Thyroid Abnormalities in Thai Patients with Vitiligo

**DOI:** 10.1155/2017/7502935

**Published:** 2017-12-07

**Authors:** Vasanop Vachiramon, Sarawin Harnchoowong, Woranit Onprasert, Kumutnart Chanprapaph

**Affiliations:** Division of Dermatology, Faculty of Medicine Ramathibodi Hospital, Mahidol University, Bangkok, Thailand

## Abstract

**Background:**

Vitiligo is an acquired hypopigmentary disorder. The prevalence of vitiligo is 0.1–2% worldwide. Numerous autoimmune diseases are associated with vitiligo, including autoimmune thyroid diseases. The prevalence of thyroid abnormalities is up to 34% in vitiligo patients depending on ethnicities.

**Objective:**

This study aims to investigate thyroid abnormalities in Thai patients with vitiligo.

**Methods:**

Medical records of vitiligo patients attending outpatient dermatology clinic at a university-based hospital from 2012 to 2016 were retrospectively reviewed. Data regarding vitiligo, clinical features, and autoimmune thyroid laboratory results were retrieved and analyzed.

**Results:**

Among 325 vitiligo patients identified, anti-thyroid peroxidase and anti-thyroglobulin were positive in 90 (27.7%) and 63 patients (19.4%), respectively. Positive thyroid antibody was associated with female gender (*p* < 0.001) and vitiliginous hand lesions (*p* < 0.02). Out of 197 patients with complete thyroid function test, the prevalence of autoimmune thyroid diseases (AITD) is 12.7%. Female, nonsegmental type, higher affected area, and the presence of leukotrichia are significantly associated with AITD in vitiligo patients.

**Conclusions:**

Prevalence of positive thyroid antibodies and AITD in Thai patients with vitiligo is compatible with previous studies around the world. Screening for AITD with thyroid antibodies and serum TSH is essential for vitiligo patients.

## 1. Introduction

Vitiligo is a common acquired pigmentary disorder. Clinical presentations are well-circumscribed depigmented macules and patches of skin and mucosa. They could occur anytime in life. However, depigmented lesions were frequently present before the age of 20 [1]. The prevalence of vitiligo varies depending on ethnicities and regions. However, it is estimated to be 0.5–2% in world population [2]. There is no preference between gender and skin types. However, female tends to develop vitiligo earlier than male [2]. The disease etiology is still unknown but autoimmune mechanism is believed to play a major role. Other pathogenetic factors include genetics, environment, and oxidative stress. These factors lead to selective loss of melanocytes [1]. Vitiligo, especially nonsegmental type, has been associated with other autoimmune disorders. Approximately 20% of vitiligo patients have at least one associated autoimmune disease. Among these, autoimmune thyroid disease (AITD) is the most common with the prevalence up to 34% in vitiligo patients [3]. However, data regarding vitiligo and thyroid abnormalities in Thai patients are limited. This study aims to explore the prevalence of positivity of thyroid antibody and AITD in Thai population and establish an association between vitiligo characteristics and thyroid abnormalities.

## 2. Materials and Methods

A retrospective study was conducted in a university-based hospital (Ramathibodi Hospital, Mahidol University, Bangkok, Thailand). The medical records of all patients with vitiligo visiting outpatient dermatologic clinic from January 2012 to December 2016 were retrospectively reviewed. Incomplete medical records were excluded. Patients with unspecified leukoderma and suspected contact leukoderma were also excluded. The study was approved from the Mahidol University Institution Review Board (IRB) for human subject research (protocol number 105941). Informed consent was exempted due to retrospective nature of the study.

We obtained demographic data of vitiligo patients including age of onset, genders, Fitzpatrick skin types, associated autoimmune diseases and underlying thyroid diseases, family history of vitiligo and autoimmune diseases, clinical features (e.g., types of vitiligo, affected body surface area, location, presence of leukotrichia, and Koebner's phenomenon or KP); anti-thyroglobulin (anti-TG), anti-thyroid peroxidase (anti-TPO), thyroid function tests, and antinuclear antibody (ANA) were recorded and filled in protocol record form.

Types of vitiligo were divided mainly into segmental, nonsegmental, and unclassified type according to revised classification of the Vitiligo Global Issues Consensus Conference [4]. KP was classified into 3 subtypes based on the Vitiligo European Task Force group [5]. KP type I was identified by history taking (i.e., vitiligo after skin trauma). KP type II was diagnosed by clinical presentation. KP type IIA is a depigmentation in the area exposed to repeated pressure and friction (elbows, knees, knuckles, etc.). KP type IIB is characterized by linear, punctiform, and crenate lesion, induced by trauma. KP type III was diagnosed by experimental induction.

Anti-TPO and anti-TG were performed by electrochemiluminescence immunoassay (Elecsys®, Roche Diagnostics GmbH, Mannheim, Germany). Tests were considered positive if anti-TPO titer > 34 IU/mL and anti-TG > 115 IU/mL. ANA was performed by indirect immunofluorescent technique (EUROPattern®, Euroimmun AG, Luebeck, Germany); a positive test was defined by titer > 1 : 80. Thyroid function test (Abbott Diagnostics, Lake Forest, IL, USA), consisting of thyroid stimulating hormone (TSH, reference range of 0.3–4.94 uIU/mL), free triiodothyronine (FT3, reference range of 1.71–3.71 pg/mL), and free thyroxine (FT4, reference range of 0.7–1.48 ng/dL), is an adjuvant investigation for further analysis. AITD were diagnosed by endocrinologists and data was included and filled in record form. Hashimoto's thyroiditis was diagnosed based on the demonstration of circulating thyroid antibodies and diffuse thyroid enlargement or reduced echogenicity on thyroid ultrasonography. The diagnosis of Graves' disease relies on persistent hyperthyroidism together with positive thyroid antibody and/or increase vascularization on thyroid sonogram. Thyroid ophthalmopathy and dermopathy are characteristic features of Graves' disease. While their presences offers diagnostic value, these are rare manifestations infrequently seen in patients.

Statistical analyses were executed by STATA statistical software version 14 (Stata Corp LP, College station, TX, USA). Genders, Fitzpatrick skin types, family history of vitiligo and autoimmune diseases, presence of leukotrichia, and KP were analyzed with standard *χ*^2^ test. While Fisher's exact test was used to compare vitiligo types and location between groups, Wilcoxon rank-sum test was used for the comparison of age at onset and the affected body surface area between each group.

## 3. Results

**Figure 1:**
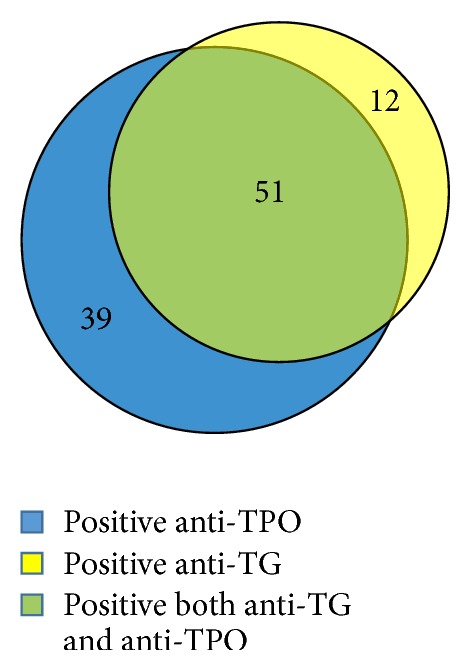
Number of vitiligo patients with positive anti-thyroglobulin (anti-TG) and anti-thyroid peroxidase (anti-TPO).

A total of 325 patients with vitiligo were included to our study. Anti-TPO and anti-TG were investigated in all patients. Cases with positivity to one or more antibody value are considered to have positive antibody. Between 325 patients, positivity to one or more thyroid antibody was found in 102 patients or 31.4%. Anti-TPO and anti-TG were positive in 90 (27.7%) and 63 patients (19.4%), respectively. Fifty-one patients had positive results to both antibodies (Figure 1). Two-hundred and twenty-three of 325 patients (68.6%) had negative thyroid antibody. Demographics and characteristics of patients with positive and negative thyroid antibody are shown in Table 1. In the group with presence of thyroid antibody, female gender was significantly predominant compared to the group with absence of thyroid antibody (77.5% versus 57%, *p* < 0.001). Median age of onset appeared to be older in the antibody positive group compared to the antibody negative group (age 40 versus 34, *p* = 0.055). Regarding location of the lesions, the presence of vitiliginous lesions on the hands was more common among patients with positive thyroid antibody compared to patients with negative thyroid antibody (37.3% versus 24.7%, *p* = 0.02), while head and neck involvement was seen more frequently among patients with negative thyroid antibody compared to patients with positive thyroid antibody (48.9% versus 33.3%, *p* = 0.009).

In our study, 53 of 325 patients (16.3%) had positive ANA. Twenty-six of 53 patients (49.1%) had fine speckled pattern. Homogeneous pattern was also seen in 26 patients (49.1%). ANA positivity was more prevalent in patients with hands (*p* = 0.009) and arms lesions (*p* = 0.048). Nevertheless, none had validation to the diagnosis of systemic lupus erythematosus (SLE).

Among 325 patients with vitiligo, thyroid function test was investigated in 197 patients. AITD was presented in 25 patients or 12.7%. Twenty-three patients were female and 2 patients were male (*p* = 0.005). Among 25 patients with AITD, 13 patients were diagnosed with Grave's disease, 8 were classified as Hashimoto's thyroiditis, and 4 had unspecified AITD. Demographic data and vitiligo characteristics of patients with and without AITD are featured in Table 2. Regarding type of vitiligo, AITD was found exclusively in nonsegmental type (*p* = 0.017). In addition, the presence of leukotrichia and higher body surface area involvement has significantly been associated with AITD (*p* = 0.035 and *p* = 0.025, resp.). Positive family history of autoimmune diseases tended to be associated with AITD development (*p* = 0.07). Vitiligo patients with Grave's disease were associated with KP type IIA (*p* = 0.01).

## 4. Discussion

Vitiligo is a common hypopigmentary disorder with unknown etiology. Genetic, environmental, and immunological factors have been implicated in the pathogenesis of this condition. However, the exact mechanism of melanocyte destruction and disease progression is still unknown.

Regarding autoimmune hypothesis of vitiligo, numerous antibodies to cell surface pigment cell antigens, intracellular pigment cell antigens, and nonpigment cell antigens have been identified in the sera of vitiligo patients [6]. Several melanocyte autoantigens have been detected in vitiligo patients including tyrosinase, tyrosinase-related protein 1, tyrosinase-related protein 2, melanosomal matrix protein gp 100 (Pmel17), melanocyte transcription factor (SOX10), and melanin concentrating hormone receptor 1 (MCHR1) [7]. Alteration in cellular immunity was also present in vitiligo [8]. Lymphocytic infiltration, dermal melanophages, degenerative changes in melanocytes, and vacuolization of basal cells were identified in normal-appearing perilesional skin and active lesions. In addition, Melan-A specific CD8+ T cells with cutaneous lymphocyte antigen have been detected in the peripheral blood of patients with vitiligo, and their number may correlate with disease activity [9–11].

Numerous autoimmune diseases in association with vitiligo have been documented in the literature including thyroid disease, diabetes mellitus, pernicious anemia, and psoriasis. Among these, autoimmune thyroid disease by far has been the most commonly reported associated condition [2]. The worldwide prevalence of positive thyroid antibodies among vitiligo patients was 2%–69% and the prevalence of AITD among vitiligo patients was 0.9%–34%, depending on type of vitiligo, ethnicities, and gender [12–14]. Our study confirms the high prevalence of thyroid antibodies and AITD among Thai vitiligo patients, which is similar to previous studies. In the present study, the prevalence of positive thyroid antibodies and AITD is 31.4% and 12.7%, respectively.

Recent discovery found genes linking generalized vitiligo to other autoimmune diseases, including TYR gene, which mediates melanin synthesis [15]. Moreover, there was evident supporting antigens crossover between vitiligo and AITD. The study analyzed expression of components in melanin synthesis pathway consisting of tyrosinase, tyrosinase-related protein 1, tyrosinase-related protein 2, lysosome-associated membrane protein 1 (LAMP1), CD 69, and some components in thyroid tissues of Hashimoto's thyroiditis patients without vitiligo. Expression of LAMP1 and CD69 in thyroid tissues of AITD patients was upregulated significantly, compared to normal thyroid tissues, while TYR was positively stained only in autoimmune thyroid tissues. These markers not only were functioning in pigmenting pathway but also were responsible for immune and oxidative stress regulation which plays a critical role in pathogenesis of autoimmune diseases. Thus, this experiment elucidates the common association between AITD and vitiligo through the mechanism of antigen crossover among the two conditions [16]. In addition, the prevalence of AITD was almost three times higher in adults compared to children (18.6% versus 6.89%, resp.) [6]. This could be explained by the fact that autoimmunity rises paralleling an age increase.

Regarding the type of vitiligo in our study, nonsegmental vitiligo is more commonly associated with positive thyroid antibody and AITD. This finding was similar to previous studies demonstrating that AITD was more commonly found in nonsegmental vitiligo [8–12], thereby confirming the autoimmune pathogenesis of nonsegmental vitiligo. Although not common, AITD can be found in segmental vitiligo as well. According to a study by Yang et al., 1 of 21 vitiligo patients with AITD had segmental type [17]. However, we could not demonstrate AITD in our patients with segmental vitiligo.

In this study, the prevalence of positive anti-TPO was 27.7% and the prevalence of positive anti-TG was 19.4%. This figure is relatively higher than the normal Thai population. Sriphrapradang et al. reported the prevalence of positive anti-TPO and anti-TG among healthy Thais to be 8.96% and 12.26%, respectively [18]. We emphasize the true significance of the high prevalence of positive thyroid antibody among our vitiligo patients.

Based on previous studies, female gender, later onset, and family history of vitiligo were risk factors in developing positive autoimmune thyroid antibodies [19–21]. The results of our study showed similar findings with previous reports. However, we additionally found that hand lesions were significantly associated with positive thyroid antibodies (*p* = 0.02) and lesions on head and neck were associated with negative thyroid antibodies (*p* = 0.009). An explanation to this could be that facial lesions are markedly noticeable leading to patients seeking early medical attention, whereas lesions on the hands may be left without cosmetic concern. Therefore, patient with vitiliginous hand lesions could have possibly had persistent disease, long enough to establish positive thyroid antibody, as mentioned earlier. Moreover, previous literature rarely evaluates anti-TG, as it is less sensitive compared to anti-TPO [22]. We evaluated both values in our study to enhance the chance to detect positive thyroid antibodies.

Female gender, longer duration of disease, higher affected body surface area, and personal and family history of other autoimmune diseases have been well-established risk factors the for the development of AITD in vitiligo patients [17, 20, 23–25]. With regard to the age, the incidence of AITD among children and adolescents with vitiligo and the control group was not significantly different [26]. However, the risk for developing AITD in vitiligo patients paralleled the age increase [12, 23]. In this study, we reported additional association between presence of leukotrichia and AITD. The presence of leukotrichia reflects long-standing disease allowing sufficient time to develop autoimmune phenomenon.

According to a study by van Geel, the presence of KP type IIA was also reported to be a risk indicator for thyroid diseases in vitiligo patients [5]. In our study, vitiligo patients with Grave's diseases were significantly featured with KP type IIA. Therefore, patients with nonsegmental vitiligo, large BSA involvement, presence of leukotrichia, and KP type IIA should be evaluated for thyroid antibodies and thyroid function test.

ANA was rarely described in vitiligo studies. Rate of ANA positivity was observed from 2.5% to 33.3% [19]. In our study, although ANA was positive in 16.3%, none were diagnosed with SLE. Therefore, ANA screening might not be useful in our vitiligo populations unless suspicious of SLE or planned for phototherapy.

The limitations of this study are retrospective design. All information and investigations may not be completed in some patients. In addition, the definite type of AITD was uncertain in some cases due to the loss to follow-up. Finally, we were unable to determine the exact timing of AITD in association to vitiligo and mark which event preceded the other.

In conclusion, we demonstrated that vitiligo is strongly associated with positive thyroid antibodies and AITD. In this study, we reported a novel association between hand lesions and positive thyroid antibodies and the presence of leuktrichia is positively correlated with AITD. Upon evaluation of vitiligo patients, physician should have high index of suspicion of associated thyroid diseases in female patients, positive family history of autoimmune disease, nonsegmental vitiligo, higher BSA involvement, and the presence of leukotrichia.

## Figures and Tables

**Table 1:** Patient's demographics and characteristics between vitiligo with positive and negative thyroid autoantibody (total 325 patients).

Data	Patients with positive thyroid antibody, *N* = 102	Patients with negative thyroid antibody, *N* = 223	*p* value
Gender, *N* (%)	Female, 79 (77.5%)	Female, 127 (57.0%)	<0.001
	Male, 23 (22.5%)	Male, 96 (43.0%)	

Fitzpatrick skin type, *N* (%)	III: 17 (16.7%)	III: 44 (19.7%)	0.26
	IV: 71 (69.6%)	IV: 161 (72.2%)	
	V: 14 (13.7%)	V: 18 (8.1%)	

Family history of vitiligo, *N* (%)	23 (22.6%)	52 (23.3%)	0.88

Family history of autoimmune diseases, *N* (%)	17 (16.7%)	22 (9.9%)	0.08

Age of onset, median (range)	40 (0–69)	34 (1–74)	0.055

Location, *N* (%)	Hand, 38 (37.3%)	Hand, 55 (24.7%)	0.02
	Head/neck, 34 (33.3%)	Head/neck, 109 (48.9%)	0.009
	Arm, 15 (14.1%)	Arm, 21 (9.4%)	0.16

Median body surface area involvement (range)	3%	2%	0.12
	(0.3–90%)	(0.1–50%)	

Type of vitiligo, *N* (%)	Nonsegmental, 87 (85.3%)	Nonsegmental, 178 (79.8%)	0.451
	Segmental, 11 (10.8%)	Segmental, 30 (13.5%)	

Leukotrichia, *N* (%)	45 (44.1%)	116 (52%)	0.186

Koebner's phenomenon, *N* (%)	IIA: 33 (32.3%)	IIA: 51 (22.9%)	0.07
	IIB: 38 (37.3%)	IIB: 79 (35.4%)	0.75
	III: 2 (2%)	III: 1 (0.5%)	0.233

**Table 2:** Patient's demographics and characteristics between vitiligo with and without autoimmune thyroid disease (total 197 patients).

Data	Patients with AITD, *N* = 25	Patients without AITD, *N* = 172	*p* value
Gender, *N* (%)	Female, 23 (92%)	Female, 111 (64.5%)	0.005
	Male, 2 (8%)	Male, 61 (35.5%)	

Fitzpatrick skin type, *N* (%)	III: 7 (28%)	III: 39 (22.7%)	0.7
	IV: 16 (64%)	IV: 122 (70.9%)	
	V: 2 (8%)	V: 11 (6.4%)	

Family history of vitiligo, *N* (%)	4 (16%)	45 (25.2%)	0.27

Family history of autoimmune diseases, *N* (%)	7 (28%)	23 (13.4%)	0.07

Age of onset, median (range)	40 (9–62)	37 (2–74)	0.84

Location, *N* (%)	Hand, 11 (44%)	Hand, 49 (28.5%)	0.11
	Head/neck, 10 (40%)	Head/neck, 77 (44.8%)	0.65
	Arm, 3 (12%)	Arm, 22 (12.8%)	1

Median body surface area involvement (range)	3% (1–80%)	2% (0.1–50%)	0.025

Type of vitiligo, *N* (%)	Nonsegmental, 25 (100%)	Nonsegmental 140 (81.4%)	0.017
	Segmental, 0 (0%)	Segmental, 23 (13.4%)	0.05

Leukotrichia, *N* (%)	18 (72%)	85 (49.4%)	0.035

Koebner's phenomenon, *N* (%)	IIA: 10 (40%)	IIA: 46 (26.7%)	0.17
	IIB: 13 (52%)	IIB: 66 (38.4%)	0.19
	III: 0 (0%)	III: 1 (0.58%)	1

AITD: autoimmune thyroid disease.
